# The Human Microbiome in Chronic Kidney Disease: A Double-Edged Sword

**DOI:** 10.3389/fmed.2021.790783

**Published:** 2022-01-17

**Authors:** Eman Wehedy, Ibrahim F. Shatat, Souhaila Al Khodor

**Affiliations:** ^1^College of Health and Life Sciences, Hamad Bin Khalifa University, Doha, Qatar; ^2^Research Department, Sidra Medicine, Doha, Qatar; ^3^Nephrology Department, Sidra Medicine, Doha, Qatar

**Keywords:** chronic kidney disease, gut microbiota, urinary microbiome, dysbiosis, uremic toxins, renoprotective, diet therapy

## Abstract

Chronic kidney disease (CKD) is an increasing global health burden. Current treatments for CKD include therapeutics to target factors that contribute to CKD progression, including renin–angiotensin–aldosterone system inhibitors, and drugs to control blood pressure and proteinuria control. Recently, associations between chronic disease processes and the human microbiota and its metabolites have been demonstrated. Dysbiosis—a change in the microbial diversity—has been observed in patients with CKD. The relationship between CKD and dysbiosis is bidirectional; gut-derived metabolites and toxins affect the progression of CKD, and the uremic milieu affects the microbiota. The accumulation of microbial metabolites and toxins is linked to the loss of kidney functions and increased mortality risk, yet renoprotective metabolites such as short-chain fatty acids and bile acids help restore kidney functions and increase the survival rate in CKD patients. Specific dietary interventions to alter the gut microbiome could improve clinical outcomes in patients with CKD. Low-protein and high-fiber diets increase the abundance of bacteria that produce short-chain fatty acids and anti-inflammatory bacteria. Fluctuations in the urinary microbiome are linked to increased susceptibility to infection and antibiotic resistance. In this review, we describe the potential role of the gut, urinary and blood microbiome in CKD pathophysiology and assess the feasibility of modulating the gut microbiota as a therapeutic tool for treating CKD.

## Introduction

Chronic kidney disease (CKD) is a growing healthcare burden affecting about 13.4% of the population worldwide (1). In the last few decades, the number of CKD patients has steadily increased (2). In adults, hypertension and diabetes are the leading causes of CKD, while congenital anomalies of the kidney and urogenital track account for the majority of CKD etiologies in children. Factors that contribute to the progression of CKD include activation of the renin–angiotensin–aldosterone system, proteinuria, a state of chronic inflammation and repetitive acute kidney injury (3–7). CKD is associated with the development of severe health conditions like cardiovascular diseases, neurological complications, adverse pregnancy outcomes, and hyperkalemia (8–12). In children, CKD affects neurocognitive abilities, school performance, growth, quality of life and the cost of medical care (6, 13–15).

Current treatments for CKD include renin–angiotensin–aldosterone system inhibitors and drugs to control blood pressure and proteinuria. An increasing number of studies suggest that the composition of the microbiome has a key role in maintaining health. The human microbiome is the collection of all microbial DNA in the human body, which is distributed in various body parts as; skin, gastrointestinal, urinary tract, respiratory tract, and oral cavity (16). These microbes play crucial roles in the digestion and metabolic processes, stimulation and regulation of the immune response, production of vitamins, and protection against pathogens (17, 18). This microbial community is in a symbiotic relation with the host in the healthy states (19). Dysbiosis refers to a disruption of the microbial balance, and this phenomenon is associated with various diseases and pathological conditions including CKD (20, 21).

**Figure 1:**
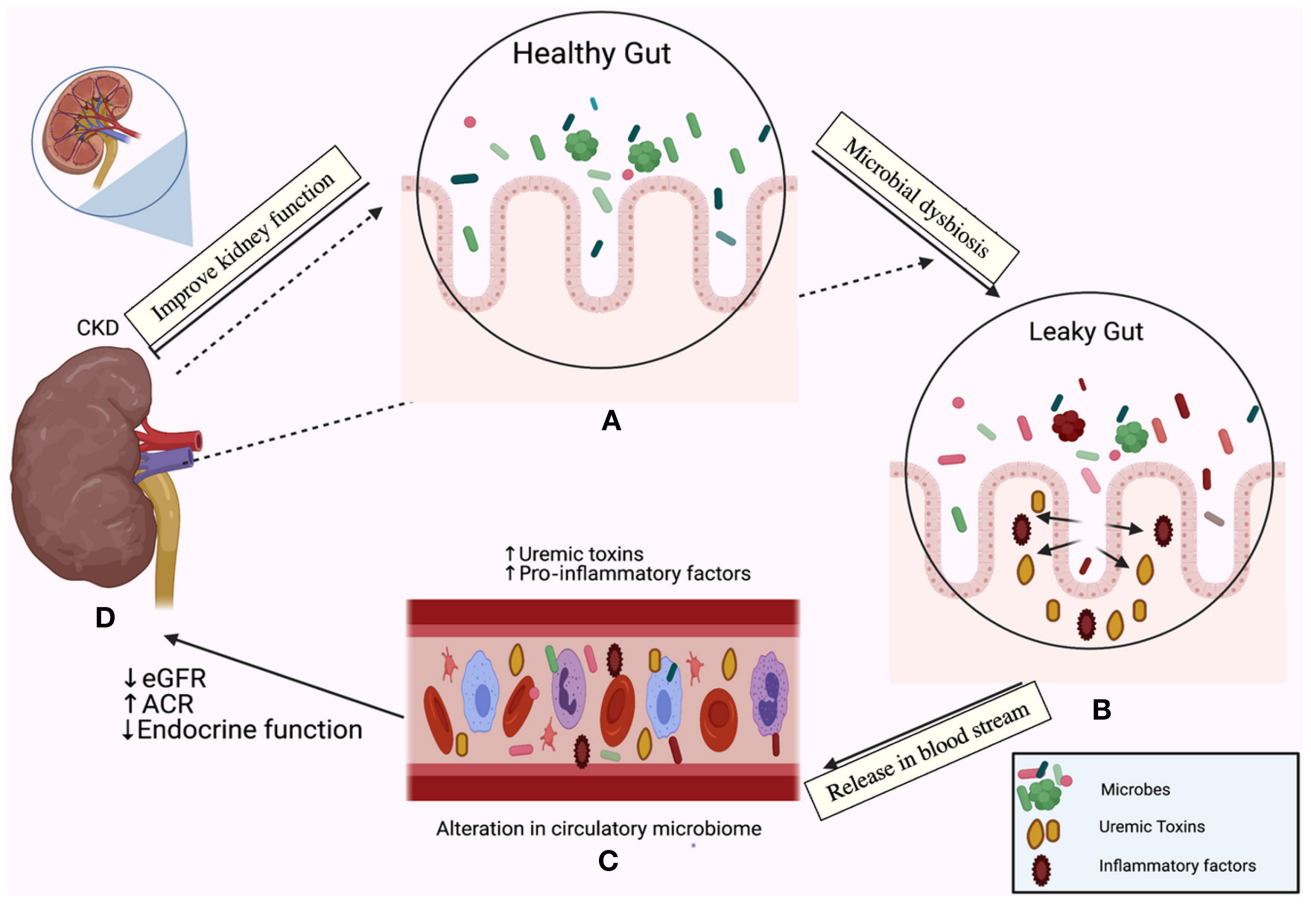
The relationship between the gut microbiome and chronic kidney disease (CKD) is bi-directional. In one direction, the gut microbiota affect the kidney; the emerging role of gut microbiota in **(A)** The healthy gut, **(B)** The leaky gut due to microbial dysbiosis and disruption of the mucosal layer, **(C)** Release of pro-inflammatory factors in the bloodstream and initiation of the inflammatory cascade, accumulation of uremic toxins, **(D)** A decline in the estimated glomerular filtration rate (eGFR), the elevation of the albumin creatinine ratio (ACR) and loss of the endocrine functions of the kidney. In the other direction, CKD drives dysbiosis in the gut (indicated by the dotted arrows) and initiates an inflammatory cascade.

The major part of the human microbiome is centralized in the gut (22). The gut microbiota harbors 10-fold more microbial cells than human cells, which regulates nutrient metabolism and produces various metabolites that affect the kidney, heart, vascular system, and liver (23). There is a bi-directional relationship between dysbiosis and the pathogenesis of CKD (20, 24–26). We summarized this relationship in Figure 1. It is well-established that an increase in levels of harmful metabolites including trimethylamine N-oxide (TAMO), indoxyl sulfate, and *p*-cresyl sulfate are associated with renal fibrosis, endothelial dysfunction, a decline in the estimated glomerular filtration rate (eGFR), cardiovascular complications, and increased mortality and morbidity in CKD (27–30). Moreover, the serum levels of 5-methoxytryptophan and indoxyl sulfate correlate positively with CKD progression (31). On the other hand, renoprotective metabolites including short-chain fatty acids prevent the progression of CKD by suppressing the disruption of the epithelial barrier and regulating the anti-inflammatory response (25, 32). The level of indole propionic acid, derived from the gut flora, negatively correlates with *p*-cresyl sulfate and indoxyl sulfate concentrations in CKD patients (33).

The composition of the gut microbiota can be altered by therapeutic dietary interventions and intake of probiotics, and so dietary interventions and probiotics intake can be used to improve CKD outcomes (27, 34). A very low-protein diet decreases plasma levels of indoxyl sulfate, and *p*-cresyl sulfate and increases the diversity of both butyrate-forming bacteria such as *Coprococcus* and *Roseburia*, and the levels of anti-inflammatory bacteria like *Blautia* and *Faecalibacterium* (27). A high-fiber diet improves kidney function by lowering the harmful uremic metabolite levels and decreasing microbial diversity (35). Conversely, a high-fat diet increases the plasma level of gut microbiota-derived TAMO metabolites in a mouse model (36).

While the gut is the main repertoire of microbes and the most studied site to date, urine and blood also harbor different types of microbes and microbial signatures in both healthy and disease conditions (22, 37, 38). Several studies have examined the role of gut-derived metabolites in CKD, but little is known about the role of the composition of the urinary and blood microbiomes in the progression of CKD (20, 28, 39).

Our manuscript will focus on the relationship between the human microbiome and its products in the CKD pathophysiology and modulating the gut microbiota as a therapeutic strategy for CKD treatment.

## Pathophysiology of CKD

A medical literature search was conducted using PubMed for articles published until May 24^th^, 2021. The initial search was done using the general search terms: “microbiome,” “chronic kidney disease,” and “metabolites.” Only articles published in English were included. The search resulted in a total of 143 articles. After excluding review articles, we focused on 57 studies summarized in Tables 1, 2, 3, we classified the studies based on the CKD model into clinical studies, animal studies, and dietary interventions studies. References of the included articles were also reviewed for additional relevant articles.

**Table 1:** Role of gut microbiota and microbial related metabolites in the pathogenesis of CKD (Clinical studies).

**CKD model**	**Microbial related metabolite (s)**	**Main findings**	**Technique(s)**	**Study**
52 ESRD patients and 44 healthy controls	Pseudouridine, l-phenylalanine, and p-hydroxyphenylacetic acid.	1. l-phenylalanine and *p*-hydroxyphenylacetic acid levels are positively correlated with CRP levels in ESRD and healthy controls groups 2. In ESRD patients, pseudouridine, l-phenylalanine, and *p*-hydroxyphenyl acetic acid metabolites positively correlated with IL-6 levels. 3. Inflammatory markers and metabolites can be used a diagnostic and predictive marker for CKD.	LC/MS	(40)
^*^223 patients with ESRD and 69 healthy controls.	PCS IS Phenylacetylglycine phenyl sulphate	1. Alteration in gut microbiota in CKD Patients; increased the abundance of Eggerthella lenta, Flavonifractor spp, Alistipes spp, Ruminococcus spp and Fusobacterium spp, and depleted Prevotella spp, Clostridium spp, SCFA-producing species and several butyrate producers (Roseburia spp, Faecalibacterium prausnitzii and Eubacterium rectale). 2. Aberrant gut microbiota and its derived uremic toxins are associated with disease development.	Shot gun metagenome sequencing and HPLC	(39)
^*^30 Caucasian CKD patients (stage 3), 121 Caucasian CKD patients (stage 5), and 80 controls	Betaine, choline, and TMAO	1. Advanced CKD patients (stage 5) expressed the lowest level of circulating betaine and highest levels of choline and TMAO compared with CKD patients (stage 3) and healthy controls. Serum betaine levels significantly declined with renal function drop.	LS-MS	(41)
103 CKD patients with stage 1 to 5 (Mild, moderate and ESRD) and 46 healthy controls	PCS, IS, TAMO, and p-cresyl glucuronide	1. CKD progression is accompanied by deterioration in kidney functions and elevation in serum uremic toxins levels. 2. The relative abundance of *Alistipes* *and Oscillibacter* is positively correlated with CKD progression, while Lachnospira*, Veillonella*, and *Dialister* negatively correlate with the disease severity. 3. *Oscillibacter* (involved in pyruvate metabolism) is positively associated with uremic toxins and CKD development.	LC-MS 16S rRNA gene sequencing	(42)
141 CKD patients and 14 controls.	PCS, IS, p-cresyl glucuronide, and Indole-3-acetic acid.	1. Increasing the plasma level of gut-derived uremic toxins is associated with impaired kidney functions and decline in eGFR. The clearance of plasma protein-bounded metabolites is decreased with disease progression.	HPLC	(28)
77 CKD patients undergoing hemodialysis and 30 healthy	Putrescine	1. Hemodialysis patients with mild cognitive decline showed a significant reduction in Class *Coriobacteriia* and genus *Tyzzerella 3, Blautia*, and Lachnospira 2. *Bilophila*, and serum putrescine (gut-related metabolite) can be a sensitive marker for cognitive decline in hemodialysis patients.	16S rRNA gene sequencing and GC-MS	(43)
72 patients with CKD And 20 controls	PCS, Secondary bile acid, and ipopolysaccharide biosynthes.	1. The early stages of CKD showed a high abundance of secondary bile acid biosynthesis microbial genes, but lipid metabolism and lipopolysaccharide biosynthesis are enriched in the advanced stages. 1. *Bacteroides eggerthii* can be a biomarker for the early stages of the disease. 2. CKD group showed a low abundance of *Prevotella sp*. 885. 3. PCS is negatively correlated with the eGFR.	Shotgun sequencing and Metabolomics (GC-MS)	(44)
115 children and adolescents with CKD stage (1–4)	TMAO, dimethylamine, and Trimethylamine	1. Gut related metabolites are associated with blood pressure abnormalities and cardiovascular risk in pediatric CKD. 2. Decrease in the diversity of Phylum *Cyanobacteria*, genera *Subdoligranulum, Faecalibacterium,* *Ruminococcus*, and *Akkermansia* in CKD patients with abnormal blood pressure. 3. Plasma levels of dimethylamine, and trimethylamine are inversely associated eGFR. Accumulation of TAMO, dimethylamine, and trimethylamine are higher in advanced CKD.	16S rRNA gene sequencing and LC–MS/MS	(24)
92 adult CKD patients (31mild, 30 moderate, and 31 advanced) and 30 healthy controls	IS and PCS	1. Accumulation of uremic toxins IS and PCS are positively correlated with disease progression. 2. The diversity of gut microbiota (genus-level: *Escherichia_Shigella,* *Dialister,* *Lachnospiraceae_ND3007_group,* *Pseudobutyrivibrio, Roseburia,* *Paraprevotella and Ruminiclostridium*, and species-level: *Collinsella stercoris* *and Bacteroides eggerthii*) are highly associated with CKD stages.	16S rRNA gene sequencing	(20)
Phase1: 10 patients with rapid decline in eGFR and 10 control patients without rapid decline Phase 2: 140 CKD and 144 healthy controls	Indole propionic acid, IS, PCS	1. Indole propionic acid produced from a healthy gut is highly reduced in CKD patients and patients with a decline in eGFR. 2. Indole propionic acid (renal protective metabolite) can be a biomarker for renal function. 3. IS and PCS were high in the CKD group compared with controls	HPLC	(33)
95 CKD patient with differed stages, 11 hemodialysis patients, and 18 healthy controls	TMAO	1. CKD patients have a high level of TMAO. Plasma TMAO is inversely correlated with eGFR.	LC-MS/MS	(29)
78 children and adolescents with CKD stage G1 to G4	Short chain fatty acids, propionate, and butyrate	1. CKD children with congenital anomalies of the kidney and urinary tract (CAKUT) showed a reduction I plasma level of propionate with increase in the relative abundance of phylum *Verrucomicrobia*, genus *Akkermansia*, and species *Bifidobacterium bifidum*. 2. The Firmicutes to Bacteroidetes ratio didn't show significant difference between groups. CKD children with an abnormal ambulatory blood-pressure monitoring profile had higher plasma levels of propionate and butyrate.	16S rRNA gene sequencing & GC-Flame Ionization detector	(45)
Eighty patients with CKD of stages 2 to 4	TMAO	1. CKD patients showed upregulation of SMAD3 and NLRP3. 2. Plasma and urine levels of TMAO are significantly high in CKD patients with elevated serum TGF-β1 and IL-1β levels. TMAO can be used as a marker for CKD progression.	Spectrophotometer	(46)
86 CKD children stage (1–3)	Urinary TMAO	1. Urinary TMAO is positively correlate *with Bifidobacterium, Lactobacillus,* *Collinsella* and *Blautia*. 2. CKD children with abnormal ambulatory blood-pressure monitoring (ABPM) profile had a lower abundance of the *Prevotella* genus than those with normal ABPM	LC-MS and 16S rRNA gene sequencing	(47)
5,469 subjects of Prevention of Renal and vascular end stage disease (PREVEND study)	TMAO	1. TMAO was positively correlated with body mass index, age and diabetes mellitus, and negatively associated with eGFR. 2. TMAO was involved in all mortality risks.	NMR	(48)
317 community-acquired pneumonia (76 CKD patients)	TMAO	1. TMAO (gut-derived) is associated with various risk factors and comorbidities. 2. The level of TMAO is reduced by antibiotic treatment (modulating gut microbiota can be a good target for reducing the TMAO risks).	LC-MS/MS	(49)
488 CKD patients (stages 1–5)	phenylacetylglutamine	1. Phenylacetylglutamine is highly accumulated in advanced stages of CKD. 2. Elevation of serum level of phenylacetylglutamine is a risk factor for cardiovascular disease high mortality rate in CKD patients.	LC-MS	(50)
51 renal transplant recipients, 51 CKD patients, and 65 stable renal transplant recipients (unrelated cohort)	PCS, IS, TMAO, p-cresyl glucuronide, and Phenylacetylglutamine	1. The serum levels of gut-derived uremic toxins are significantly decreased after renal transplantation compared with CKD patients.	UPLC-MS/MS	(51)
227 CKD patients (had cardiovascular surgery for coronary artery disease)	TMAO	1. The advanced stage of CKD showed the highest TMAO level. 2. The elevated level of TMAO is associated with CKD development and infracted coronary arteries.	HPLC-APCI -MS/MS	(52)
^*^20 CKD on HD and 60 healthy controls (Divided into 3 group 20 controls each)	p-cresol and indole (precursors of PCS and IS)	1. CKD is highly affected by microbial metabolism. 2. The dietary intervention significantly impacts microbial metabolism (CKD patients and healthy household controls on the same diet have a similar microbial metabolism pattern).	GC-MS	(53)
^**^521 stable subjects with CKD	TAMO	1. TAMO is associated with the progression of renal dysfunction, poor prognosis, and mortality risk in CKD patients. 2. Elevated TMAO is a risk factor for cardiovascular disease.	LC/MS/MS	(54)

* *The study also included in Table 2*;

** *The study also included in Table 3. IS, Indoxyl sulfate; PCS, p-cresyl sulfate; TMAO, trimethylamine N-oxide; LC, liquid chromatography; GC, gas chromatography; MS, mass spectrometer; HPLC, high performance liquid chromatography; eGFR, estimated glomerular filtration rate; ESRD, end stage renal disease; SCFAs, short chain fatty acids; NMR, nuclear magnetic resonance*.

**Table 2:** Role of gut microbiota and microbial related metabolites in the pathogenesis of CKD (Animal models studies).

**CKD Model**	**Microbial related metabolite (S)**	**Main findings**	**Technique(s)**	**Study**
Organic anion transporter 1 (Oat1) knockout mice and wild type	IS, kynurenine, and xanthurenic acid	1. Oat1 knockout mice expressed accumulation of IS, kynurenine, and xanthurenic acid metabolites. 2. There is a remote link between gut microbiota, phase II metabolism in the liver and the elimination of uremic toxins in the kidney through Oat1.	LC-MS/MS	(55)
Male C57BL/6 mice with unilateral nephrectomy	IS	1. Accumulation of circulatory uremic toxin (IS) was associated with neurological complications and increased oxidative stress in the CKD mice model. 2. Applying AST-120 (Uremic toxin adsorbent) reversed the effects of IS. 3. Gut microbiota is a potential target to control CKD progression and its related circumstances.	HPLC	(56)
Male C57/BL6 mice (Unilateral ureteral obstruction and sham control)	Myo-inositol, dodecanoic acid, N-acetylputrescine, and anthranilic acid.	1. CKD mice group showed a reduction in short-chain fatty acids-producing genera (Bacteroides, Prevotellaceae_UCG-001, Roseburia, and Lachnospiraceae_NK4A136_group). 2. Bacteroides and Prevotellaceae_UCG-001were negatively associated with renal fibrosis. 3. Parasutterella and Alistipes were positively correlated with CKD progression. 4. Myo-inositol, dodecanoic acid, N-acetylputrescine, and Anthranilic acid were positively correlated with renal damage.	16S rRNA gene sequencing and LC-MS	(57)
^*^Different animal species; Free-ranging brown bears (Ursus arctos), Captive brown bears, Garden dormice, Captive lions and tigers, Wild boars (Susscrofa)	betaine, choline, and TMAO	1. Dietary mode (carnivores and omnivores) significantly affects betaine, choline, and TMAO levels. 2. Circulating betaine levels were at the highest level in active dormice. 3. Active, free-ranging brown bears showed the lowest level of choline, while the highest choline levels were found in active garden dormice. 4. In one out of 15 free-ranging bears, TMAO was detectable, although it was detected in all captive bears.	LC-MS	(41)
Female Sprague-Dawley rats (Maternal CKD model)	TAMO & SCFAs	1. CKD Rats showed a high accumulation of TAMO, reduction in SCFAs (acetate, butyrate), and dysregulation of the renin-angiotensin system. 2. CKD group expressed a higher Firmicutes to Bacteroidetes ratio compared to the controls group. 3. Phylum Bacteroidetes was low abundance in the CKD group. Maternal CKD is associated with hypertension and renal damage in the adult male offspring.	16s rRNA gene sequencing and LC-MS	(58)
Male IQI, C57BL/6Njc1, and C57BL/6JJcl mice	Purine, Allantoin, and purine	1. Germ-free mice showed renal damage and inflammatory responses with high expression of purine metabolizing enzymes. 2. Purine metabolizing enzymes are responsible for converting adenine to a nephrotoxic byproduct 2,8-dihydroxyadenine.	CE-TOF/MS	(59)
^*^ Germ free adenine-induced CKD mice (transplanted with fresh gut microbiota from ESRD patients and healthy controls. & 5/6 nephrectomy CKD rat.	PCS, phenylacetylglycine, phenyl sulphate, and IS	1. PCS, phenylacetylglycine, phenyl sulphate and IS, were significantly increased in mice that received ESRD microbiota. 2. Aberrant gut microbiota is responsible for renal disease development. 3. High abundance of *Eggerthella lenta* and *Fusobacterium nucleatum* species in CKD model, but gavage the rat with *Bifidobacterium animalis* A6 decreased their abundance.	16S rRNA gene sequencing and HPLC	(39)
Subtotal nephrectomy rat model	Gut microbiome-derived uremic solutes	1. There is a complex communication between gut, kidney, blood, and liver controlling uremic toxin retention. 2. Organic anion transporters (OATs) are essential to evaluate plasma uremic toxins levels.	UPLC-MS	(60)
5/6 nephrectomized (NX) rat model and sham controls.	Glycine-conjugated compounds and polyamine metabolites.	1. CKD rats expressed a high abundance of *Allobaculum,* *Escherichia_Shigella,* *Clostridium_sensu_stricto,* *Bacteroides, Parasutterella,* *Ruminococcus, Blautia* and *Enterorhabdus*. 2. *Blautia* was positively correlated with proteinuria. 3. The creatinine clearance rate is affected by gut flora and polyamine metabolism.	1.16S rRNA gene sequencing and UPLC–MS	(61)
Germ free and specific pathogen free IQI Mice	SCFAs, Indole-3-acetic acid, and n-3 type of polyunsaturated fatty acid	1. Gut-derived metabolites (SCFAs) have a preventive role in the disruption of endothelial barriers. Germ-free mice expressed significantly low SCFAs with renal failure.	LC-MS/MS	(25)
Male Sprague-Dawley rats (Unilateral ureteral obstruction (UUO) and sham operating control)	Tryptophan related metabolites (kynurenine, 5-hydroxytryptophan and 5-hydroxytryptamine)	1. Plasma levels of tryptophan-related metabolites were high in the UUO group and showed a positive association with tubulointerstitial fibrosis. 2. UUO changed the abundance of some gut microbiota (reduction and enrichment). 3. Clostridium IV, Turicibacter, Pseudomonas, and Lactobacillales were positively correlated with plasma tryptophan level, while Oscillibacter, Blautia, and Intestinimonas showed a negative correlation.	16S rRNA gene sequencing and UPLC-MS	(62)
Chronic nephropathy rats and controls rats	Hydrogenated and demethylated loganetin, demethylated morronisid aglycone and dehydroxylated morronisid aglycone	1. The gut microflora of healthy rats is more efficient in degrading loganin and morronisid than that of nephropathy rats.	UPLC-Q-TOF/MS	(63)
Adenine-induced renal failure and control mice (with germ-free or specific pathogen-free conditions)	Uremic toxins as (IS, PCS, TAMO, choline) & SCFAs	1. Gut microbiota has a dual role in CKD, it is responsible for the accumulation of uremic toxins, but the germ-free mice model showed severe renal damage. 2. Shortage of SCFAs and reduction in amino acid metabolism were higher in germ-free mice than specific pathogen-free mice.	CE-TOF/MS	(26)
Male Sprague–Dawley rats CKD mode	Quercetin glycosides, myricetin and gossypetin.	1. Flos A. Manihot extract (herbal extract) affected microbial metabolism, especially on deglycosylation and methylation metabolic pathways. 2. CKD model samples showed a lower concentration of quercetin, myricetin, and gossypetin than control samples.	UPLC-Q-TOF/MS	(64)
^*^Wistar rats 5/6th nephrectomy and sham operated control rats	p-cresol and indole (precursors of PCS and IS)	1. Accumulation of uremic toxins are higher in CKD group than in control group. 2. Gut-derived metabolites (IS and PCS) are associated with CKD progression, cardiovascular disease risk, and overall mortality.	GC-MS	(53)
Male Sprague-Dawley rats (5/6 nephrectomy and sham operation control)	Isoprene, laldehydes and thioesters.	1. The CKD rats group showed changes in the gut-related metabolites profile in the exhaled breath and gaseous gut microbiota products.	GC	(65)

* *The study also included in Table 1, IS, Indoxyl sulfate; PCS, p-cresyl sulfate; TMAO, trimethylamine N-oxide; CE-TOF/MS, capillary electrophoresis time-of-flight mass spectrometry-based approach; HPLC, high performance liquid chromatography; LC, liquid chromatography; GC, gas chromatography; MS, mass spectrometer; LC, liquid chromatography; GC, gas chromatography; MS, mass spectrometer; HPLC, high performance liquid chromatography; eGFR, estimated glomerular filtration rate; ESRD, End stage renal disease; SCFAs, short chain fatty acids; UPLC, ultraperformance liquid chromatography*.

**Table 3:** Effect of dietary intervention on CKD outcomes.

**CKD model**	**Dietary regimen**	**Microbial related metabolite (s)**	**Main findings**	**Technique(s)**	**Study**
C57BL/6J male mice NPX or sham operation and ApoE^−/−^ mice (B6.129P2- Apoe^tm1Unc^/J)	Three groups for 2 weeks **Group 1:** High fat diet (HFD) + Vehicle **Group 2:** HFD + unconjugated p-cresol (uPC) **Group 3:** HFD + PCS **Fecal transplantation** *ApoE–/–* mice fed with HFD and fecal transplantation from CKD mice and Sham Control Mice for 19 weeks (3 times a week).	Unconjugated p-cresol (uPC) and PCS	1. Mice fed with HFD and had fecal transplantation showed increased coronary artery lipids deposits. 2. Non-CKD mice treated with uPC expressed a higher level of total cholesterol, and triglycerides, apoptosis. 3. Mice fed a high-fat diet (atherosclerosis) develop higher coronary artery lipid deposits after receiving fecal material from CKD mice.	LC-MS	(66)
Male Sprague-Dawley (SD) rats	Six groups (4 weeks intervention) N group (normal rats) M group (CKD rats) HK group (Huangkui capsule treatment) RR group (Rehmanniae Radix Preparata treatment) CF group (Corni Fructus treatment) RC group (RC treatment)	Acetamidovalerate, glutamine, phenyllactic acid, tryptophan, 7-ketolithocholic acid and deoxycholic acid.	1. Treatment with Rehmanniae Radix and Corni Fructus showed an increase in the relative abundance of beneficial gut microbiota (*Ruminococcaceae UCG-014,* *Ruminococcus 1,* *Prevotellaceae_NK3B31_group,* *Lachnospiraceae NK4A136* group, and Lachnospiraceae UCG-001). 2. Fecal metabolites (Acetamidovalerate, glutamine, phenyllactic acid, tryptophan, 7-ketolithocholic acid, and deoxycholic acid), involved in amino acid metabolism, bile acids metabolism and glycerophospholipid metabolism, were associated with CKD.	16S rRNA gene sequencing and UPLC-Q-TOF -MS/MS	(67)
C57BL/6 mice	**Diet experiment (28 days)** Group 1: Normal chow + Folic acid nephropathy (FAN) Group 2: High Fiber diet+ FAN **SCFAs Experiment** Group 1: Sodium Acetate + FAN Group 2: Propionate + FAN Group 3: Butyrate + FAN Group 4: Control water + FAN	SCFAs	1. The High fiber-diet mice group expressed less tubular injury and higher kidney protection than control group on day 2 and on day 28 showed less chronic inflammation and interstitial fibrosis. 2. The High fiber diet and SCFAs supplementation increased the relative abundance of Bifidobacterium and Prevotella (SCFA-producing bacteria).	16S rRNA gene sequencing and ^1^H NMR spectroscopy	(68)
43 CKD undergo peritoneal dialysis (26 completed the follow-up)	2 groups for 12 weeks **Group 1:** Unripe banana flour (UBF−48% resistant starch for 4 weeks > washout for 4 weeks > placebo (waxy corn starch) for 4 weeks. **Group 2:** Placebo for 4 weeks > washout for 4 weeks > UBF for 4 weeks.	IS, PCS, and indole 3-acetic acid (IAA)	1. The serum levels of uremic toxins (IS, PCS, and indole 3-acetic acid) did not express any significant change by UBF prebiotic treatment. 2. IL-6 was slightly higher after UBF treatment.	HPLC	(69)
Female C57BL/6J (apoE KO mice)	14 weeks before tissue collection: **Group 1:** Chow diet (control) **Group 2**: Control + 0.06% iodomethylcholine (IMC) **Group 3:** Control + 0.2% adenine **Group 4:** Control + 0.2% adenine + 0.06% IMC	TMAO and TMA	1. Adenine diet is associated with CKD progression, increasing plasma level of TAMO, and inducing myocardial hypertrophy. 2. Supplementation of IMC in group 2 and group 3 showed improvement of renal injury and decreased plasma TAMO than in group 1 and group 3, respectively. 3. Controlling the TAMO level can be a targeted treatment for CKD.	LC-MS	(70)
Female Virgin Sprague-Dawley rats (Maternal adenine induced CKD)	3 groups for 18 weeks **Group 1 (Control):** Regular diet 3 weeks > regular diet for 6 weeks (pregnancy and lactation) > Regular diet (Male offspring till 3 months age). **Group 2 (CKD):** Control diet + adenine for 3 weeks > regular diet for 6 weeks > Regular diet (Male offspring till 3 months age). **Group 3 (CKD+R):** Control diet + adenine for 3 weeks > Regular diet + resveratrol for 6 weeks > Regular diet (Male offspring till 3 months age).	Plasma TMA, plasma TMAO and fecal SCFAs	1. Resveratrol treatment showed a protective effect against hypertension in the adult male offspring of maternal CKD. 2. Resveratrol beneficially modifies the gut microbiota by increasing the abundance of the genera *Lactobacillus* and *Bifidobacterium* *and decreasing the* *Firmicutes* to *Bacteroidetes* ratio. 3. The plasma TMA significantly increased in the resveratrol treated group than the CKD group with decreasing TMAO-to-TMA ratio. 4. The male offspring's kidneys expressed increasing G-protein coupled receptor-41 (GPR41) protein levels in the resveratrol treated group.	16s rRNA gene sequencing and LC-MS	(71)
C57BL6 Mic 5/6 nephrectomy CKD-induced and healthy controls	Four groups (4 weeks) **Group 1:** Control mice + regular diet. **Group 2:** Control mice + high fiber diet (Resistant starch). **Group 3:** CKD-induced mice+ regular diet. **Group 4:** CKD-induced mice + high fiber diet.	NA	1. Healthy mice fed with a resistance starch (HRS) diet showed upregulation in tryptophan and indole metabolism compared with CKD and CDK-RS groups. 2. RS diet reduced CKD progression compared with regular diet in CKD mice. 3. RS increased the abundance of beneficial bacteria (butyrate-producing bacteria) and decreased mucin-degrading bacteria. 4. *Oscillibacter sp*. 1–3 was the most abundant bacterium in healthy mice fed with RS	de novo sequencing using PEAKS	(72)
43 CKD patients and 34 matched normal non-CKD controls	Three groups (3 months) **Group 1:** Low protein diet (LPD) CKD patients. **Group 2:** normal protein diet (NPD) CKD patients. **Group 3:** Non-CKD controls.	SCFAs, IS, PCS	1. Butyrate-producing bacteria (family *Lachnospiraceae* and *Bacteroidaceae*) markedly decreased in the CKD-LPD group with a lowering in the serum SCFAs levels (acetic, heptanoic, and non-anoic acid) and a significant increase in glyco λ-muricholic acid (secondary bile acid). 2. The three groups did not express any significant changes in IS and PCS serum levels.	16S rRNA gene sequencing and GC-MS	(73)
28 CKD dogs and 28 healthy dogs	Six groups (30 weeks interventions **Group 1:** Control food (10 weeks) > Low soluble fiber plus betaine food (10 weeks) > High soluble fiber plus betaine food (10 weeks). **Group 2:** Control food (10 weeks) > High soluble fiber plus betaine food (10 weeks) > Low soluble fiber plus betaine food (10 weeks).	SCFAs and uremic toxins	1. CKD dogs expressed a lower level of SCFAs and higher levels of gut-related uremic toxins, creatinine, urea, and indoles than healthy controls. 2. The low soluble fiber plus betaine food significantly reduced genus *Collinsella* of (phylum Actinobacteria) compared with the control food and the high soluble fiber plus betaine food.	16S rRNA gene sequencing & LC-MS	(74)
	**Group 3:** Low soluble fiber plus betaine food (10 weeks) > Control food (10 weeks) > High soluble fiber plus betaine food (10 weeks). **Group 4:** Low soluble fiber plus betaine food (10 weeks) > High soluble fiber plus betaine food (10 weeks) > Control food (10 weeks). **Group 5:** High soluble fiber plus betaine food (10 weeks) > Control food (10 weeks) > Low soluble fiber plus betaine food (10 weeks). **Group 6:** High soluble fiber plus betaine food (10 weeks) > > Low soluble fiber plus betaine food (10 weeks) > Control food (10 weeks).		3. The highest abundance of Phylum Bacteroidetes (genus *Odoribacter*) was in the low soluble fiber plus betaine food, followed by the control food, and the high soluble fiber plus betaine food had the lowest abundance. CKD dogs consuming low soluble fiber plus betaine and the high soluble fiber plus betaine foods expressed a healthier profile than CKD dogs fed with control food.		
Wistar rats (5/6 nephrectomy and sham controls)	Two groups (8 weeks) **Normal diet group** For nephrectomy rats (Nx) and sham control rats **Normal diet** **+** **paramylon group** For nephrectomy rats (Nx +PAR)	Tricarboxylic acid cycle-related metabolites (cis-aconitic acid, citric acid, isocitric acid, and malic acid)	1. Nxand Nx + PAR groups exhibited a lower abundance of *Lactobacillus* and *Rothia* and a higher abundance of *Clostridium* than the sham control group. 2. Paramylon supplementation (Nx+ PAR) significantly increased the abundance of *Eubacterium*, Marvinbryantia, *Parvibacter, Coprococcus, Robinsoniella*, and *Bacillus* compared with NX with a regular diet. 3. Paramylon improved renal function (reduce tubulointerstitial injury and glomerulosclerosis) and inhibited the accumulation of uremic toxins like tricarboxylic acid cycle-related metabolites and gut-derived metabolites.	16S rRNA gene sequencing & CE-TOFMS	(75)
CKD cats and healthy controls cats	**Pre-trial food:** Diet® k/d® Feline with chicken, dry for 14 days **Group 1(Food A for 4 weeks):** Pre-trial food + betaine (0.500%) + oat beta glucan (0.586%) + 0.407% scFOS. **Group 2 (Food B for 4 weeks):** Pre-trial food + betaine (0.500%) + oat beta glucan (0.586%) + 3.44% apple pomace	Creatinine, urea, and some microbial and host tryptophan metabolites (indole sulfates and kynurenate).	1. The plasma levels of creatinine, urea, indole sulfates, and kynurenate were higher in CKD cats than controls at the baseline of the interventions. 2. CKD cats-Food B group showed higher levels of more oxidized glutathione and inflammatory sphingolipid metabolites than CKD cats-Group A. 3. The relative abundance of *Bacteroidales* was higher in CKD cats-Food A than in CKD cats at the baseline.	16S rRNA gene Sequencing & LC-MS & GC-MS	(76)
Virgin Sprague–Dawley rats (Maternal CKD model)	**Pregnancy intervention (3 weeks)** Group 1: Control Mice + Vehicle Group 2: Control Mice + Tryptophan Group 3: CKD + Vehicle Group 4: CKD + Tryptophan	Nitric oxid-related metabolites, including l-citrulline (the precursor of l-arginine), l-arginine	1. The relative abundance of the genera Lactobacillus and Ruminiclostridium_9, decreased in maternal CKD, while genus Ruminococcus_1 increased. 2. Male offspring of maternal CKD rats expressed hypertension, but tryptophan supplementation during pregnancy prevented it.	16S rRNA gene sequencing and HPLC	(77)
	**Male offspring intervention (12 weeks)** Group 1: Vehicle (CN) Group 2: CKD Group 3: Tryptophan supplementation (Trp) Group 4: CKD + Tryptophan supplementation (CKDTrp)		3. Increasing phylum Firmicutes and reduction of Bacteroidetes were associated with maternal tryptophan supplementation. 4. Trp and CKDTrp interventions increased the Firmicutes to Bacteroidetes ratio compared with the control Group. 5. Tryptophan is thought to prevent hypertension by controlling nitric oxide and the renin-angiotensin system pathways and modulating tryptophan-metabolizing microbes.		
Male C57BL/6JJcl mice	4 Groups (4 weeks intervention) ** Group 1:** Control Mice + Vehicle ** Group 2:** Control Mice + AST-120 (carbon absorbent) ** Group 3:** Renal failure (RF) + Vehicle ** Group 4:** Renal failure + AST-120	IS & PCS	1. The RF Group had decreased *Bacteroidetes* and increased *Firmicutes* compared to the control. 2. AST-120 treatment affected the PCS and IS levels, the relative abundance on gut microbiota, and metabolic enzymes associated with p-cresol and tryptophan production. 3. RF mice treated with AST-120 expressed an increase in the relative abundance of *Faecalibaculum, Blautia* and *Desulfovibrio*, and a decrease in the relative abundance of Roseburia.	16 S rRNA gene Sequencing and GC-MS	(78)
Male C57BL/6J mice	Four Groups (5 weeks intervention) ** Group 1 (control):** Standard normal rodent diet without choline ** Group 2:** Control diet + TMAO ** Group 3:** Control diet + choline ** Group 4:** Control diet + choline + iodomethylcholine (IMC) All Groups treated with isoproterenol after one week of the interventions for 28 days.	TMAO and its precursor choline	1. TMAO and choline diets were associated with renal functions impairment and renal fibrosis, while IMC supplementation showed significant improvement in renal functional metrics. 2. The relative abundance of *Bacteroides* was lower in the TAMO and choline Groups than in the control Group, and the addition of IMC reversed this effect. 3. Group 2 and Group 3 diets had a marked enrichment of *Lachnospiraceae_UCG-002* with a positive correlation of Cystatin C and ACR and a negative correlation with GFR.	16S rRNA gene sequencing and LC–MS/MS	(79)
60 CKD patients (grades 3B-4), randomly assigned for two Groups.	** Group 1:** Free diet (3 months) > very low protein diet (6 months) > free diet (3 months) > Mediterranean diet (6 months). ** Group 2:** Free diet (3 months) > Mediterranean diet (6 months) > free diet (3 months) > very low protein diet (6 months).	IS PCS	1. The uremic toxins (IP and PCS) levels were negatively correlated with very low protein and Mediterranean diets. 2. Very low protein and Mediterranean diets significantly increased butyrate-forming bacteria (*Lachnospiraceae, Ruminococcaceae, Prevotellaceae*, and *Bifidobacteriaceae)* and reduced *Enterobacteriaceae* (harmful). 3. The very low protein diet showed enrichment of Blautia and Faecalibacterium, Coprococcus, and Roseburia species (beneficial modulation of gut microbiota).	16s rRNA gene sequencing and LC-MS/MS	(27)
21 CKD patients on hemodialysis patients (10 Placebo Group and 11 Probiotic Group)	**Placebo** **Group:** Three capsules (wheat germ) per day for 3 months **Probiotics** **Group:** Three capsules (*Streptococcus thermophilus* (KB19), *Lactobacillus acidophilus* (KB27), and *Bifidobacteria longum* (KB31)) Per day for 3 months.	TMAO and TAMOA precursors (choline, betaine)	1. Probiotic supplementation didn't affect the TMAO level but significantly increased the betaine level. 2. In the placebo Group, the choline level was increased.	LC-MS/MS	(34)
Sprague–Dawley rats	Salviae Miltiorrhizae Radix et Rhizoma (SMR) treatment (4 weeks). Group A: Normal rat model Group B: Chronic renal failure rat model	Dihydrotanshinone I and miltirone	1. Gut microbiota Mucispirillum, Kurthia, Clostridium, Blautia, Butyrivibrio, Shuttleworthia, Peptococcus, Ruminococcus, Bradyrhizobium, Methylobacterium, Azospirillum, Thalassospira, Methylophilus, Pseudomonas, peptostreptococcaceae and bacteroidales showed a significant change after SMR treatment. 2. The modulatory effect of SMR on the gut microbiota is thought to be promoted by the bioactive materials (tanshinones and salvianolic acids).	16s rRNA gene sequencing UPLC-QTOF/MS	(80)
Sprague–Dawley rat (5/6 nephrectomy CKD and sham controls)	Four Groups for 8 weeks interventions ** Group 1:** Sham rats + vehicle (tap water) ** Group 2:** Sham rats + 3,3 dimethyl-1-butanol (DMB) ** Group 3:** CKD rats + vehicle ** Group 4:** CKD rats + DMB	TAMO	1. The serum level of TAMO was significantly high in the CKD-Vehicle Group compared with the sham-vehicle Group. 2. TAMO is associated with elevation of proinflammatory cytokines and oxidative stress. 3. DMB (TMAO inhibitor) partially neutralize the effect of TAMO.	LC-MS	(81)
Male C57BL/6 mice	Four Groups for 16 weeks interventions ** Group 1:** High fat diet + controls ** Group 2:** High Fat diet + 3,3 dimethyl-1-butanol (DMB) ** Group 3:** Low fat Diet + controls ** Group 4:** Low fat diet + DMB	TAMO	1. The high-fat diet was positively associated with the elevation of plasma level of gut-derived TAMO. 2. Mice fed with a high fiber diet showed renal interstitial fibrosis, obesity induction, metabolic alteration, and increased expression of pro-inflammatory cytokines compared with a low-fat diet. 3. Combining DMB (TAMO inhibitor) to the high-fat diet showed improvement of TAMO circumstances.	LC-MS/MS	(36)
Male Sprague-Dawley rats (CKD and cecectomized rats	Two Groups (24 weeks intervention) ** Group 1:** CKD rats ** Group 2:** CKD rats + AST-120 treatment (carbon absorbent)	IS and phenyl sulfate	1. Uremic toxins (IS and phenyl sulfate) in urine and serum were higher in the CKD rats compared with the control rats. 2. CKD and CKD + AST-120 rats showed lower diversity and richness than control rats. 3. Treatment with AST-120 decreased the uremic toxins levels. 4. Cecectomized rats expressed a change in the uremic toxins production and gut microbiota composition (production depends on endogenous gut microbiota). 5. Indole and phenol-producing gut bacteria (belongs to *Clostridia* and *Bacteroidia*) significantly reduced the relative abundance in cecectomized rats.	LC-MS and 454-pyrosequencing of the 16S rRNA gene	(82)
Male Sprague–Dawley rats Sham controls and Uremia (CKD-induced)	Three Groups (4 weeks) ** Group 1:** Sham controls ** Group 2:** Uremia ** Group 3:** Uremia + Probiotic (*Lactobacillus LB*) All Groups were fed with standard diet.	Fecal metabolites	1. CKD-induced rats showed alteration in the fecal compared with controls; Lactobacillus intervention partially overturned these changes. 2. The fecal docosahexaenoic acid, 3-(3-hydroxyphenyl) propionic acid, phenethylamine glucuronide, stearoyl serotonin, and aspartyl-glutamine metabolites were significantly decreased after probiotic treatment.	UPLC-MS	(83)
Male Sprague-Dawley rats (adenine-induced CKD)	Group 1: Semipurified low-fiber diet for 3 weeks. Group 2: High-fiber diet Resistant starch **(**HAMRS2) for 3 weeks.	IS, PCS	1. The high fiber diet increased decreased the serum and urine levels of IS and PCS metabolites. 2. Rats fed with a high fiber diet showed an increase in the Bacteroidetes-to-Firmicutes ratio than those fed with a low fiber diet. 3. The high fiber diet was positively correlated with the relative abundance of *Actinobacteria* and *Proteobacteria*.	16s rRNA gene sequencing and GC-TOF/MS	(35)
40 CKD patients	Two Groups for 12 weeks ** Group 1:** prebiotic arabinoxylan oligosaccharides (AXOS) (10 g twice daily) and maltodextrin for 4 weeks > washout (4 weeks) > Placebo (potato starch) 4 weeks. ** Group 2:** Placebo for 4 weeks > washout (4 weeks) > AXOS and maltodextrin for 4 weeks	PCS, IS, TMAO, p-cresyl glucuronide, and phenylacetylglutamine.	1. Gut related uremic toxins and insulin resistance did not show any significant change after prebiotic treatment.	UPLC—MS/MS	(84)
^*^C57BL6J mice	Three diet Groups for 6 weeks ** Group 1:** Chemically defined diet (standard chow diet) ** Group 2:** Standard chow diet supplemented with choline. ** Group 3:** Standard Chow d diet supplemented with TAMO. Another study for 14 weeks to evaluate cystatin C level.	TAMO	1. Dietary choline and TAMO are responsible for progressive renal tubulointerstitial fibrosis and renal injury. 2. Gut-derived TAMO has a causative role in CKD progression.	HPLC	(54)

* *The study also included in Table 1, IS, Indoxyl sulfate; PCS, p-cresyl sulfate; TMAO, trimethylamine N-oxide; TMA, trimethylamine; SCFAs, short chain fatty acids; NA, not applicable; GFR, glomerular filtration rate; LC, liquid chromatography; GC, gas chromatography; MS, mass spectrometer; HPLC, high performance liquid chromatography; UPLC, ultraperformance liquid chromatography; TOF-MS, time-of-flight mass spectrometry-based approach*.

To sum up three studies were included in the clinical (Table 1) and animal (Table 2) studies, and one study was included in Tables 1, 3 as different CKD models have been used in these four studies. In the tables, we focused on the techniques used to assess gut microbiota and microbial related metabolites not all the techniques used in each study. Of note, gut microbiota and its related metabolites have a significant role in CKD progression and are associated with different circumstances. The exact mechanism of the gut-kidney axis is not clearly identified and both mechanisms of the role of gut microbiota in CKD (causative or consequence) are applicable. Furthermore, dietary intervention studies using animal models showed a change in the disease outcomes, but in humans did not show a big difference. This may be due to most of the participants were in a late CKD stage (ESRD or CKD on hemodialysis) in which most kidney functions are compromised.

CKD encompasses a spectrum of pathophysiologic processes associated with abnormal kidney function and a progressive decline in the glomerular filtration rate (GFR) (29). The underlying etiology varies by age, presence of co-morbid conditions, repeated occurrences of acute kidney injury and level of proteinuria (5, 6). The decline in kidney function and micro-structural changes are considered chronic when they last more than 3 months (85). Irrespective of the underlying etiology (which is considered the initiating mechanism), hyperfiltration and hypertrophy of the remaining nephrons, tubulointerstitial fibrosis, activation of the renin–angiotensin–aldosterone system, and disruption of endothelial barriers disruption are common and lead to a reduction in the renal excretion efficacy and decline in the eGFR (3, 86). The eGFR is used to grade disease severity in CKD patients, a higher grade is associated with a lower filtration rate and more advanced disease (85). The transition from one grade to the next grade is usually accompanied by a loss in the endocrine function of the kidney (86). In particular, CKD patients suffering from cardiovascular events show deterioration in renal functions and severe inflammation (87). Infiltration of immune cells in the tubulointerstitial space and accumulation of immune-derived components contribute to CKD progression (88). A key goal of CKD therapies is to prevent patients progressing to the next stage of the disease.

## Microbial Dysbiosis in CKD

The dominant bacterial phyla in the gut are Firmicutes, Bacteroidetes, Actinobacteria, and Proteobacteria (89). The interplay between bacteria present in the gut (and their metabolites) and kidney function is occasionally referred to as the gut–kidney axis (90). Recent studies indicate that aberrant gut microbiota has a key role in the pathophysiology of CKD with severe CKD outcomes (24, 61). *Bifidobacterium* and *Lactobacilli* are negatively correlated with CKD progression and long-term survival (27, 34, 39). A study of 223 patients with end-stage renal disease revealed that *Eggerthella lenta*, Fusobacterium nucleatum, and Alistipes shahii are positively correlated with increased levels of secondary bile acids and uremic toxins in CKD patients compared with the control group (39). In this study, the authors showed that the presence of Faecalibacterium prausnitzii, Roseburia, and Prevotella (which produce short-chain fatty acids) was negatively correlated with disease progression and uremic toxin accumulation (39). Another study of 92 patients with CKD reported an increased abundance of *Paraprevotella, Pseudobutyrivibrio, and Collinsella stercoris* in the CKD cohort; this finding led the authors to suggest that this signature can be used to discriminate between patients with CKD (even those in the early stages of the disease) and healthy individuals (20).

Dysbiosis in the gut has an emerging role in many inflammatory-related diseases and is thought to contribute to the inflammatory component of both acute and chronic kidney disease (91, 92). Microbial alterations in the gut affects the permeability of the intestinal mucosal barrier and releases pro-inflammatory factors and endotoxins in the bloodstream, which initiate the inflammatory cascade (93).

Another mechanism by which gut dysbiosis may contribute to CKD progression is via the role of gut dysbiosis in endothelial dysfunction, the vasoconstrictor response, and the subsequent development of hypertension; a well-known risk factor for CKD (94, 95). Mice fed a high-salt diet had aberrant microbiota compared with mice fed a normal diet; these changes were associated with activation of T-lymphocytes and an elevation in blood pressure (96). A lower abundance of *Lactobacillus* species in the gut is associated with the development of hypertension and kidney diseases (97). Changes in the gut microbiota could be the starting point for CKD progression through a series of immune response modifications, blood pressure alterations, metabolic changes, and prolonged inflammation.

## Microbial Metabolites in CKD

**Figure 2:**
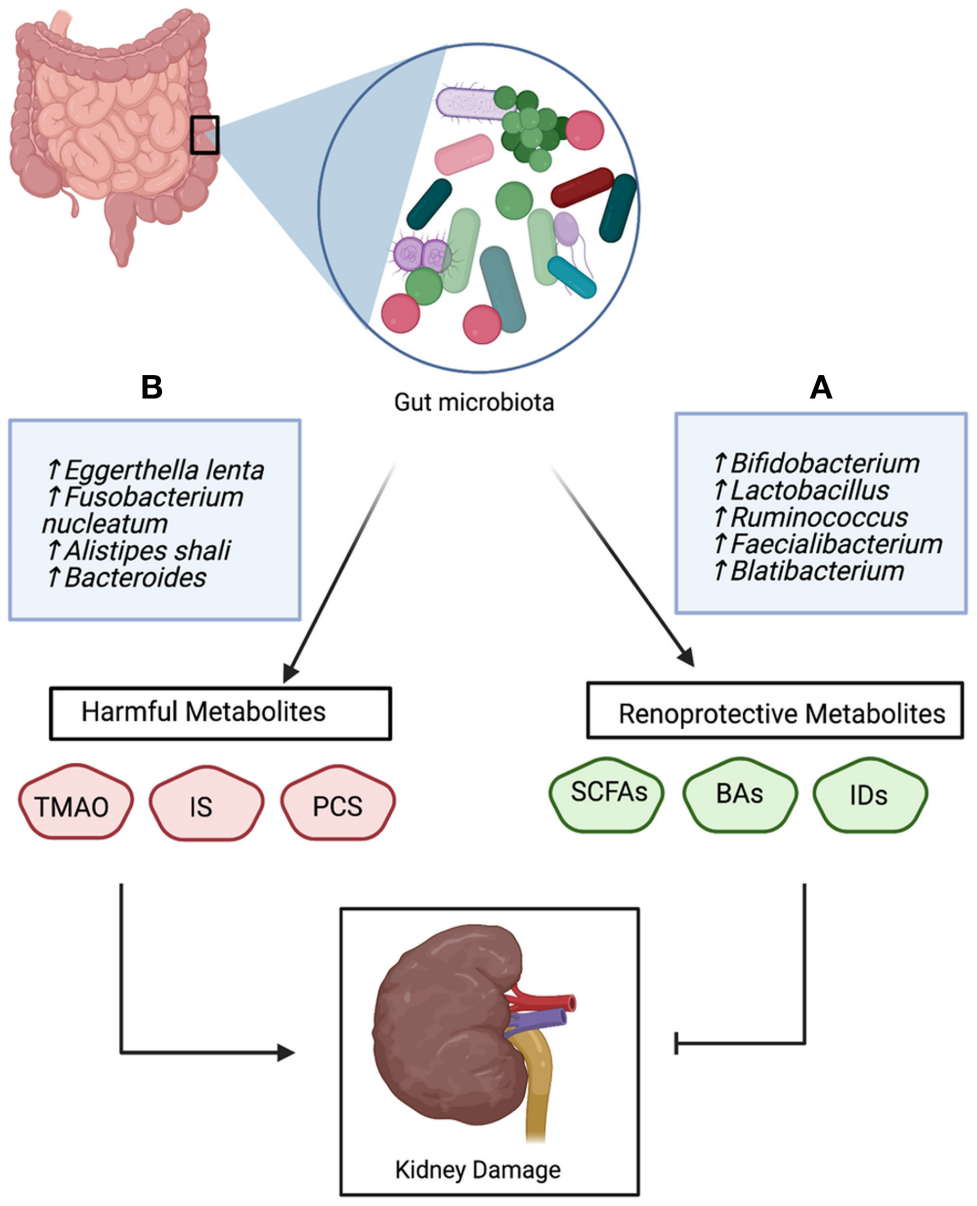
The bidirectional role of gut-derived metabolites in the pathophysiology of CKD; **(A)** Beneficial bacteria produce renoprotective metabolites that inhibit kidney damage, **(B)** Unfavorable bacteria produce harmful metabolites which promote kidney damage and CKD progression. Trimethylamine N-oxide (TMAO), indoxyl sulfate (IS), p-cresyl sulfate (PSC), short-chain fatty acids (SCFAs), bile acids (Bas), and Indole derivatives (IDs).

In general, microbial metabolites associated with CKD are classified into two groups; harmful and renoprotective metabolites. This bi-direction relationship of microbial-derived metabolites is illustrated in Figure 2. Several human and animal studies have demonstrated the deleterious effects of TAMO on the kidney, manifested as kidney interstitial fibrosis, eGFR decline, endothelial dysfunction, and an increased risk of cardiovascular disease risk (34, 36, 47, 81). The increased risk of mortality and morbidity in patients with CKD has been attributed to the accumulation of indoxyl sulfate and *p*-cresyl sulfate (27, 28, 33, 39). These toxins bind with high affinity to plasma proteins, which mitigates their removal through the dialysis membrane (28). TAMO, indoxyl sulfate, and *p*-cresyl sulfate are involved in SMAD signaling, tryptophan metabolism, and tyrosine pathways, respectively (20, 46, 54).

A wide array of uremic toxins and other microbial metabolites accumulate in biological samples of patients with CKD, including those in common biological samples such as plasma, stool, and urine, but also volatile metabolites in exhaled breath and gases collected from fecal cultures. For example, accumulation of gaseous metabolites including isoprene, aldehyde, dimethyldisulfide, dimethyltrisulfide, and thioesters occurs in patients with CKD (65).

## Dietary Interventions and CKD

Microbial dysbiosis and accumulation of gut-derived metabolites have been reported in CKD patients (20, 29, 53). Randomized controlled clinical trials in patients with CKD indicate that changes in the composition of the gut microbiota after treatment with prebiotics and probiotics improved disease outcomes and reduced uremic toxin levels (98–100). Patients with a high abundance of *Bifidobacterium* and *Lactobacillus* had lower serum levels of uremic toxins, a reduced inflammatory milieu, and improved renal function (98, 101).

The consumption of food items rich in choline and L-claritin—which are precursors of TAMO—such as egg yolk, kidney, liver, meat and milk, correlates with a high accumulation of uremic toxin and a decline in the glomerular filtration rate (102). A prospective, crossover clinical trial randomized 60 patients with CKD to different dietary interventions; the group on the very low-protein diet had an increase in the gut abundance of *Actinobacteria* and a decrease in the inflammatory *Proteobacteria* compared with the group on a regular diet (27).

Prebiotics are non-digestible dietary components such as dietary fiber and digestion-resistant starch. They are present in cereals, fruits, milk, honey, and vegetables or can be given as a dietary supplement (103). Fermentation of prebiotics beneficially modifies gut bacteria by increasing the abundance of *Bifidobacterium spp* and *lactobacillus* and reducing the levels of *Bacteroides, Clostridia*, and *Enterobacteria* (104). In patients with CKD, dietary fiber intake decreases the levels of circulating pro-inflammatory cytokines, slows the decline in eGFR, lowers the plasma levels of uremic toxins, and minimizes CKD-related cardiovascular risk (105, 106). Esgalhado and colleagues studied the effect of digestion-resistant starch supplementation (16 g/day) in patients with CKD; they observed a reduction in the plasma levels of uremic toxins (indoxyl sulfate, and *p*-cresyl sulfate), interleukin (IL)-6, and thiobarbituric acid-reactive substances (107). These results are consistent with another study of 32 patients with CKD randomized into two groups; the group that received lactulose syrup for 8 weeks had a greater abundance *Bifidobacterium* and *Lactobacillus* in the gut microbiome and decreased serum creatinine levels (108). While these studies demonstrated that probiotics and prebiotics have a beneficial effect on CKD, other studies have shown no significant changes in circulating gut-derived metabolites or changes in CKD outcomes (34, 84). It is important to point out that existing studies are heterogeneous; they used different dietary supplements, had varying durations of intervention, and administered to patients with other comorbidities, patients with varying kidney disease severity and varying underlying etiology. This heterogeneity makes it extremely difficult to draw conclusions from these studies. That being said, superior results may be obtained from the study of dietary interventions in children as other cofounding factors are minimal.

Overall, these studies imply that nutrition therapy has the potential to modulate the microbiome composition and its metabolites, and consequently ameliorate CKD complications and the rate of CKD progression. However, further well-designed, prospective studies are needed to definitively demonstrate the benefit of nutrition therapy on CKD.

## Role of the Urinary and Blood Microbiomes in CKD

Most of the attention in the microbiome field is on the gut microbiota and its metabolites; however, the urinary microbiome is receiving more attention. Until recently, urine was considered a sterile fluid that was only rendered unsterile because of infection (109, 110). But the development of next-generation sequencing techniques enabled studies showing that the urinary tract of healthy individuals is dominated by different kinds of microbes, and the distribution pattern of these microbes affects the health of urinary tract health (110, 111). Fluctuation in the urinary microbiome occurs in urinary tract infections and is involved in antibiotic resistance (109, 112). The urinary microbiome undergoes changes after kidney transplantation, and these modifications thought to be responsible for allograft dysfunction and increased susceptibility to infection (113, 114). In addition, the diversity in microbes in the urinary tract of patients with CKD is associated with the eGFR value (115).

The circulatory microbiome in healthy individuals contains diverse bacterial taxa, and the dominated phylum is Proteobacteria (38). Gut-derived endotoxins circulating in the bloodstream were shown to alter the blood microbiome (93). A study investigating the correlation between blood metabolome and α-diversity of gut microbiota on 399 participants indicated that gut-derived metabolites like p-cresyl and TAMO reflect the Shannon diversity of gut bacteria and could be a biomarker reflecting the gut health (116). A case-controlled study using 16S rRNA target sequencing of blood samples showed that compared with control groups, patients with CKD had a higher diversity of *Enterobacteriaceae* and *Pseudomonadaceae*, which was also correlated with lower eGFR (117). Hence, we see the gut microbiota has an ultimate effect on CKD outcomes through different routes.

## Conclusion

Microbial dysbiosis plays an important role in the pathogenesis of various diseases. In this review, we summarized and reviewed the literature examining the dual role of the gut microbiome and its metabolic products in the pathophysiology and progression of CKD. We described how gut dysbiosis can initiate the inflammatory process and cause leaking of gut-derived metabolites into the bloodstream. It is well-established that TAMO, indoxyl sulfate, and *p*-cresyl sulfate and other harmful microbial metabolites accumulate in patients with CKD, and levels of these metabolites correlate with disease progression. Lower levels of *bifidobacterium, lactobacillus*, and bile acid composition are linked to adverse outcomes in patients with CKD. Our analysis of the literature suggests that the complex interaction between the gut, urinary tract and blood microbiota and associated metabolites may orchestrate subclinical changes in the pathogenesis of CKD and contribute to disease. Modulating the gut microbiota using dietary interventions could improve the clinical outcomes of patients with CKD. Our recommendations are i. conducting omics-based studies like metagenomics and metatranscriptomics to identify the gut microbiota community, metabolic pathways, and microbial genes associated with CKD. ii. screening gut microbiota at different disease stages, especially at the early disease stages. iii. performing dietary intervention studies for CKD patients in the early stages. iv. assessment of urinary and blood microbiome studies for CKD patients. These directions may give a clue about the disease etiology, metabolic pathways and potential treatment for CKD.

## Author Contributions

EW wrote the first draft. IS and SA reviewed and finalized the content of the manuscript. All authors read and approved the final version.

## Funding

This work is funded by Sidra Medicine Internal Research Fund 2019 (No. SDR 200055).

## Conflict of Interest

The authors declare that the research was conducted in the absence of any commercial or financial relationships that could be construed as a potential conflict of interest.

## Publisher's Note

All claims expressed in this article are solely those of the authors and do not necessarily represent those of their affiliated organizations, or those of the publisher, the editors and the reviewers. Any product that may be evaluated in this article, or claim that may be made by its manufacturer, is not guaranteed or endorsed by the publisher.
